# A Novel Algorithm for Imbalance Data Classification Based on Neighborhood Hypergraph

**DOI:** 10.1155/2014/876875

**Published:** 2014-08-11

**Authors:** Feng Hu, Xiao Liu, Jin Dai, Hong Yu

**Affiliations:** Chongqing Key Laboratory of Computational Intelligence, Chongqing University of Posts and Telecommunications, Chongqing 400065, China

## Abstract

The classification problem for imbalance data is paid more attention to. So far, many significant methods are proposed and applied to many fields. But more efficient methods are needed still. Hypergraph may not be powerful enough to deal with the data in boundary region, although it is an efficient tool to knowledge discovery. In this paper, the neighborhood hypergraph is presented, combining rough set theory and hypergraph. After that, a novel classification algorithm for imbalance data based on neighborhood hypergraph is developed, which is composed of three steps: initialization of hyperedge, classification of training data set, and substitution of hyperedge. After conducting an experiment of 10-fold cross validation on 18 data sets, the proposed algorithm has higher average accuracy than others.

## 1. Introduction

The imbalanced dataset problem in classification domains occurs when the number of instances that represent one class is much larger than that of the other classes. The minority class is usually more interesting from the point of view of the learning task. There are many situations in which imbalance occurs between classes, such as satellite image classification [1], risk management [2], and medical diagnosis [3, 4]. When studying problems with imbalanced data, using the classifiers produced by standard machine learning algorithms without adjusting the output threshold may well be a critical mistake [5]. At present, the solutions for the problem of imbalanced dataset classification are developed at both the data and algorithmic levels [6]. At the data level, the objective is to rebalance the class distribution by resampling the data space, such as oversampling the minority class and undersampling the prevalent class. At the algorithm level, solutions try to adapt existing classifier learning algorithms to strengthen learning with regard to the minority class, such as cost-sensitive learning, ensemble learning, and hypernetwork [7].

Previous research improved resampling methods in many aspects and proposed some effective resampling algorithms. SMOTE is an intelligent oversampling algorithm that was proposed by Chawla et al. [8]. Its main idea is to form new minority class samples by interpolating between several minority class samples that lie together. Thus, the overfitting problem is avoided and the decision space for the minority class spread further; meanwhile, it reduces the decision space for the majority class, so many researchers proposed different improved methods. Dong and Wang [9] proposed the Random-SMOTE, which is different from SMOTE, which obtained new minority class samples by interpolating among three minority class samples. Yang et al. [10] proposed ASMOTE algorithm which chose not only the minority class samples but also the majority class samples that are near to minority class sample, avoiding synthetic sample overlapping the majority class samples. Han et al. [11] proposed the Borderline-SMOTE. Hu and Li [12] proposed NRSBoundary-SMOTE algorithm which can expand the decision space for the minority class; meanwhile, it will shrink the decision space for the majority class.

While in recent years, with the rapid developing of ensemble methods for classification, they have been applied to imbalanced data classification, ensemble learning is a machine learning paradigm where multiple learners (called base learners) are trained to solve the same problem [13]. Due to the outstanding performance of ensemble methods, they are applied to imbalanced dataset by combining with other techniques. Chawla et al. have developed SMOTEBoost algorithm by integrating Adaboost (the most famous boosting algorithm) and synthetic minority oversampling technique (SMOTE) [14]. Similarly to SMOTEBoost, RUSBoost also introduces data sampling into the Adaboost algorithm, while it applies random undersampling to the majority class; but SMOTEBoost creates synthetic new minority class instances by operating in the feature space [15]. Błaszczyński et al. integrate a selective data preprocessing method SPIDER with Ivotes ensemble algorithm developing the framework called IIvotes [16]. Besides, cost-sensitive learning becomes an effective tool to solve class imbalanced problem, which involves two types: binary classification problem and multiclassification problem. It can be implemented by two ways that are rescaling and reweighted, respectively. Both of them aim at making the trained classification algorithms cost sensitive. Rescaling changes the distribution of samples in training data. It has been used in cost-based sampling [17], REBALANCE [18], Rescalenew [19], and so on. Differing from rescaling, reweighted adjusts the class probability distribution in classifier based on costs. It has been used in MetaCost [20] proposed by Domings and AdaCost [21], which is improved by Fan et al. according to AdaBoost.

In the 1970s, Rumelhart and Norman proposed three types of human learning: accretion, tuning, and restructuring [22]. Based on their study, professor Zhang proposed three basic principles of cognitive learning [23]: (1) continuity, (2) glocality, and (3) compositionality. He used a hypergraph as presentation form and proposed a hypernetwork model, which can be used for cognitive learning and memory. Hypernetwork is a probabilistic graph with numbers of hyperedges. A hyperedge can be regarded as a component, a subject, or even a circuit. From the perspective of data, a hyperedge is the combination of sample attributes and class. So far, hypernetwork can just deal with discrete data. Dataset must be discrete before using for building a hypernetwork classifier. A hypernetwork model includes three steps: (1) initializing of a hypernetwork model according to training dataset, (2) evolutionary learning of a hypernetwork, and (3) classification of test dataset using the evolutionary hypernetwork. In step (1), a sample is used to generate many hyperedges through inheriting some attributes of the sample and its class. In step (2), operations of match, selection, and replacement are repeatedly executed for hyperedges. It is started from a randomly initialized hypernetwork; in each iteration, fitness of a hyperedge is calculated for evaluating and ordering. Hyperedges which own low fitness are replaced with new generated hyperedges. In this way, hyperedges with high class discernibility in pattern space can be found out by the hypernetwork [7]. After the above steps, a hypernetwork model is built and classifies the test data through joint probability.

Although hypernetwork has been widely used in solving various machine learning problems, it usually produces poor overall classification performance when dealing with class imbalance problems. Like most of the traditional classification algorithms, hypernetwork assumes that the class distribution of datasets is balanced. The goal of the hypernetwork learning is to extract hyperedges (or decision rules) that can cover as many samples as possible. Hyperedges are critical for differentiating class membership which are copied and added while hyperedges with poor differential ability are discarded. However, within the context of class imbalance, many samples in minority class are usually viewed as noises. Therefore, the number of hyperedges corresponding to the majority significantly surpasses that of hyperedges corresponding to the minority. As a result, most of the minority samples are misclassified in a traditional hypernetwork. Thus, this paper attempts to combine hypernetwork with rough set to address the problem.

Rough set theory is a powerful mathematical tool introduced by Pawlak [24–27] to deal with imprecise, uncertain, and vague information. It has been successfully applied to such fields as machine learning, data mining, intelligent data analysis, and control algorithm acquiring. Basically, the idea is to approximate a concept by three description sets, namely, the lower approximation, upper approximation, and boundary region. The rough set theory puts the uncertain samples in the boundary region and the boundary region can be calculated by upper approximation minus lower approximation, and they all can be calculated. Until now, there are many researchers who brought rough set theory to process imbalanced data [28, 29].

The remainder of this paper is organized as follows. The basic concepts on neighborhood rough set models are shown in Section 2. The neighborhood hypergraph algorithm is developed in Section 3.  Section 4 presents the experimental evaluation on 18 imbalanced UCI datasets [30] by 10-fold cross validation, which shows the validity of the proposed method. The paper is concluded in Section 5.

## 2. Hypergraph and Neighborhood Hypergraph Model

### 2.1. The Definition of Hypergraph

In 1970, Berge [31] used hypergraph to define hypernetwork. And it was the first time to establish undirected hypergraph theory systematically and it was applied on the operations research by matroid.

**Definition 1 (see [31, 32]).**

Given *V* = {*v*_1_, *v*_2_,…, *v*_*n*_} is finite set, if

**Figure 1:**
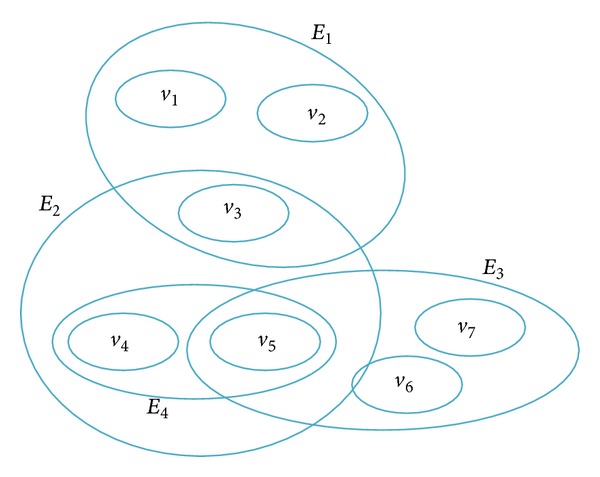
An example of hypergraph.

- *E*_*i*_ ≠ *ϕ*  (*i* = 1,2,…, *m*),
- ⋃_*i*−1_^*m*^ *E*_*i*_ = *V*,

then the binary relation *H* = {*E*, *V*} is defined as a hypergraph. The elements *v*_1_, *v*_2_,…, *v*_*n*_ of *V* are defined as vertices of the hypergraph; *E* = {*E*_1_, *E*_2_,…, *E*_*m*_} is defined as the edge set of hypergraph. *E*_*i*_ = {*E*_*i*_1__, *E*_*i*_2__,…, *E*_*i*_*j*__} is defined as hyperedge (see Figure 1).

### 2.2. The Neighborhood Hypergraph

Neighborhoods and neighborhood relations are a class of important concepts in topology. Lin [33] pointed out that neighborhood spaces are more general topological spaces than equivalence spaces and introduced neighborhood relation into rough set methodology. Hu et al. [34] discussed the properties of neighborhood approximation spaces and proposed the neighborhood-based rough set model. And then they used the model to build a uniform theoretic framework for neighborhood based classifiers. For the convenience of description, some basic concepts of the neighborhood rough set model are introduced here at first.

**Definition 2 (see [34]).**

Given arbitrary *x*_*i*_ ∈ *U* and *B*⊆*C*, the neighborhood ∂_*B*_(*x*_*i*_) of *x*_*i*_ in the subspace *B* is defined as

(1) $$\begin{array}{c} \partial_{B}\left(x_{i}\right)=\left\{x_{j}\;|\;x_{j}\in U,\Delta_{B}\left(x_{i},x_{j}\leq \delta \right)\right\}, \end{array}$$

where Δ is a metric function. ∀*x*_1_, *x*_2_, *x*_3_ ∈ *X* ∪ *E*, it satisfies

1. Δ(*x*_1_, *x*_2_) ≥ 0,
2. Δ(*x*_1_, *x*_2_) = 0 if and only if *x*_1_ = *x*_2_,
3. Δ(*x*_1_, *x*_2_) = Δ(*x*_2_, *x*_1_),
4. Δ(*x*_1_, *x*_3_) ≤ Δ(*x*_1_, *x*_2_) + Δ(*x*_2_, *x*_3_).

Consider that *x*_1_ and *x*_2_ are two objects. *A* = {*a*_1_, *a*_2_,…, *a*_*m*_} is a sample-dimensional space, where *f*(*x*, *a*_*i*_) denotes the value of sample on the *i*th dimension *a*_*i*_. Then, a general metric, named Minkowsky distance, is defined as

(2) $$\begin{array}{c} \Delta_{p}\left(x_{1},x_{2}\right)={\left(\overset{m}{\underset{i=1}{\sum }}{\left|f\left(x_{1},a_{i}\right)-f\left(x_{2},a_{i}\right)\right|}^{p}\right)}^{1/p}. \end{array}$$

When *P* = 2, it is the Euclidean distance Δ_2_.

But Euclidean distance can only be used to compute continuous features; the nominal features are invalid. Here, we compute them by using value difference metric (VDM) proposed by Stanfill and Waltz [35] in 1986. The distance between two corresponding feature values is defined as follows:

(3) $$\begin{array}{c} f\left(x_{1},V_{1}\right)-f\left(x_{2},V_{2}\right)=\overset{n}{\underset{i=1}{\sum }}{\left|\frac{C_{1i}}{C_{1}}-\frac{C_{2i}}{C2}\right|}^{k}. \end{array}$$

In the previous equation, *V*_1_ and *V*_2_ are the two corresponding feature values. *C*_1_ is the total number of occurrences of feature value *V*_1_ and *C*_1*i*_ is the number of occurrences of feature value *V*_1_ for class *i*. A similar convention can be also applied to *C*_2*i*_ and *C*2. *k* is continuous, which is usually set to 1.

**Definition 3 .**

Given *G* = 〈*X*, *E*〉 is a neighborhood hypergraph, then *X* = {*x*_1_, *x*_2_,…, *x*_*n*_} is the vertex set of *G*, indicating that it has *n* vertices, where *x*_*i*_ denotes a sample. *E* = {*e*_1_, *e*_2_,…, *e*_*n*_} is hyperedge set, and each *e*_*i*_ in *E* is a hyperedge which connects *k* vertices (*x*_*i*1_, *x*_*i*2_,…*x*_*ik*_). *C* = {*c*_1_, *c*_2_,…, *c*_*m*_} is the attribute set, and *D* is the decision set.

**Figure 2:**
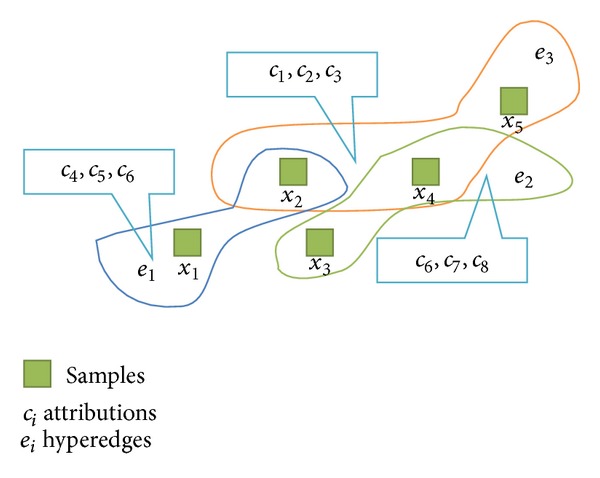
An example of neighborhood hypergraph.

Vertices of hypergraph represent the attribution of samples in some literatures like literature [36] and so on. However, in this paper, vertices of hypergraph are denoted as samples and different samples on one hyperedge have the same attributes set. An example of neighborhood hypergraph is as in Figure 2.

**Definition 4 .**

Given *x* = {*c*_1_(*x*), *c*_2_(*x*), *c*_3_(*x*),…, *c*_*n*_(*x*), *D*(*x*), *δ*} is a sample, where *c*_1_(*x*), *c*_2_(*x*), *c*_3_(*x*),…, *c*_*n*_(*x*) denote the values of *x* at the attributes set *C*, *D*(*x*) denotes the decisions of *x*, and *δ* is the radius of a neighborhood.

**Definition 5 .**

Given *G* = 〈*X*, *E*〉 and the attribute set *C*, the hyperedges which are included by sample *x*, consisting of the set of hyperedges, are defined as

(4) $$\begin{array}{c} \delta_{C}\left(x\right)=\left\{e\;|\;\left(e\in E\right)\wedge \Delta \left(e,x\right)\leq \delta \right\}. \end{array}$$

**Definition 6 .**

Given *G* = 〈*X*, *E*〉 and attributes set *B*  (*B*⊆*C*), for arbitrary *e* ∈ *E*, the sample set *in*_*B*_(*e*) which related to *e* is defined as *in*_*B*_(*e*) = {*x*∣*e* ∈ *δ*(*x*), *x* ∈ *X*}. Given arbitrary *Y*⊆*E* and attributes set *B*(*B*⊆*C*), the sample set *in*_*B*_(*Y*) related to *Y* is defined as *in*_*B*_(*Y*) = {*in*_*B*_(*e*)∣*e* ∈ *Y*}.

**Definition 7 .**

Given *G* = 〈*X*, *E*〉, for arbitrary *e* ∈ *E*, one knows *D*(*e*)∈{*P*, *N*}, where *P* denotes minority decision and *N* denotes majority decision. Then sets of decision *P* and decision *N* in hyperedge set *E* are, respectively, defined as

(5) $$\begin{array}{c} nE\left(P\right)=\left\{e\;|\;D\left(e\right)=P,e\in E\right\},\\ nE\left(N\right)=\left\{e\;|\;D\left(e\right)=N,e\in E\right\}; \end{array}$$

thus, the degree of imbalance for *E* is defined as

(6) $$\begin{array}{c} inbanE=\frac{\left|nE\left(N\right)\right|}{\left|nE\left(P\right)\right|}. \end{array}$$

**Definition 8 .**

Given *G* = 〈*X*, *E*〉, for arbitrary *e* ∈ *E*, assume *D*(*e*)∈{*P*, *N*}, where *P* denotes minority decision and *N* denotes majority decision. *in*_*B*_(*e*) is sample set related to hyperedge *e* on attributes set *B*⊆*C*. According to decisions *D*, *in*_*B*_(*e*) is divided into *p* equivalence classes: *X*_1_, *X*_2_,…, *X*_*p*_; when *in*_*B*_(*e*) ≠ *ϕ*, the confidence degree of *e* is defined as follows.

(1) If *D*(*e*) = *P*, then
  (7) ConfB(e) =|{x ∣ x∈inB(e),D(x)=D(P)}|×inbanE  ×(|{x ∣ x∈inB(e),D(x)=D(N)}|+|{x ∣ x∈inB(e),D(x)=D(P)}|×inbanE)−1.
(2) If *D*(*e*) = *N*, then
  (8) ConfB(e) =|{x ∣ x∈inB(e),D(x)=D(N)}|  ×(|{x ∣ x∈inB(e),D(x)=D(N)}|+|{x ∣ x∈inB(e),D(x)=D(P)}|×inbanE)−1.

**Definition 9 .**

Given *G* = 〈*X*, *E*〉, *C* is the attitudes set of samples, and *D* is the samples decision. For arbitrary hyperedge set *E*′  (*E*′⊆*E*), according to decisions *D*, the hyperedge set *E*′ is divided into *p* equivalence classes: *E*_1_, *E*_2_,…, *E*_*p*_. For arbitrary *B*⊆*C*, the upper approximation, lower approximation, boundary region, and negative domains of decision *D* related to set of attributes *B* are, respectively, defined as

(9) $$\begin{array}{c} {\bar{N}}_{B}\left(D\right)=\overset{p}{\underset{i=1}{⋃}}\left\{e\;|\;in_{B}\left(e\right)\cap in_{B}\left(E_{i}\right)\neq \phi \vee \left(e\in E_{i}\right),e\in E^{\prime }\right\},\\ {\underline{N}}_{B}\left(D\right)=\overset{p}{\underset{i=1}{⋃}}\left\{e\;|\;\text{Con}{\text{f}}_{B}\left(e\right)=1,e\in E_{i}\right\},\\ BN_{B}\left(D\right)={\bar{N}}_{B}\left(D\right)-{\underline{N}}_{B}\left(D\right),\\ \text{Ne}{\text{g}}_{B}\left(D\right)=E-{\bar{N}}_{B}\left(D\right). \end{array}$$

The lower approximation of decision *D* that related to the set of attributes *C* is also called positive domain, denoted by POS_*B*_(*D*). The size of positive domain reflects the separable degree of classification problem in a given attribute space; the bigger the positive region, the smaller the border.

**Figure 3:**
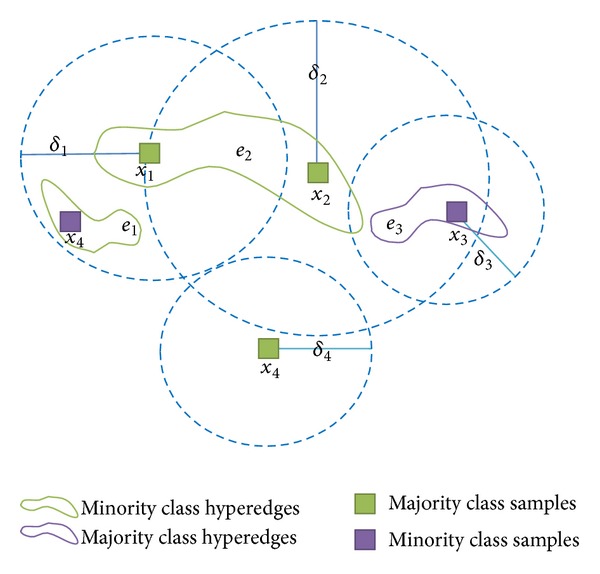
An example of upper, lower approximation, and boundary region.

To explain how to divide the upper approximation, lower approximation, and boundary region, here we give an example (Example 1) in Figure 3.

In Figure 3, the hyperedge *e*_2_ is simultaneous in the neighborhood of samples *x*_1_ and *x*_2_; in other words, it links *x*_1_ and *x*_2_. From the graph, we can know easily that whether a hyperedge is in the neighborhood is up to the fact that whether the symbol *e*_*i*_ of the hyperedge is inside of the neighborhood of the sample.

First, one calculates the sample set *in*_*B*_(*e*_*i*_) of each hyperedge: *in*_*B*_(*e*_1_) = {*x*_1_}, *in*_*B*_(*e*_2_) = {*x*_1_, *x*_2_}, *in*_*B*_(*e*_3_) = {*x*_2_, *x*_3_} according to Formula (4) and Definition 6. Second, one calculates the confidence degrees of each hyperedge, according to Formula (8): Conf_*B*_(*e*_1_) = 0, Conf_*B*_(*e*_2_) = 1, Conf_*B*_(*e*_3_) = 0.5. Third, according to Formula (9), one gets the final result on Figure 3: the upper approximation ${\bar{N}}_{B}(D)=\{e_{1},e_{2},e_{3}\}$, the lower approximation ${\underline{N}}_{B}(D)=\{e_{2}\}$, and the boundary region *BN*_*B*_(*D*) = {*e*_1_, *e*_3_}.

## 3. Neighborhood Hypergraph Classification Algorithm

Traditional hypernetwork model has limit on some aspects as follows: (1) discretized datasets. (2) There is no special processing to the samples in boundary region. However, some advantages will appear when rough set theory is combined with hypernetwork: (1) hypernetwork can directly deal with numeric data, which avoids information loss of data. (2) In the process of hyperedge learning, hyperedge set is divided into three parts that are upper approximation, lower approximation, and boundary region. In addition, hyperedges in boundary region will be processed specially, which will result in the improvement of classification accuracy.

The proposed algorithm aims at tackling imbalanced data classification problem including two aspects as follows.

(1) Improve the degree of imbalance of hyperedge set. The class of traditional hyperedge is inherited from samples directly, which is helpless to improve the degree of imbalance of hyperedge set. In the paper, when initializing hyperedges, classes of fractional hyperedges are determined according to the classes of samples, which reduces the degree of hyperedge set to some extent.

(2) Set classification condition. The classification process of traditional Hypernetwork does not take the degree of hyperedge set into consideration, resulting in a low accuracy of minority class. However, one sets a threshold, which equals the square of the degree of imbalance, as a classification condition. This method makes the classifier pay more attention to minority class and thus can deal with class imbalance problem appropriately.

**Figure 4:**
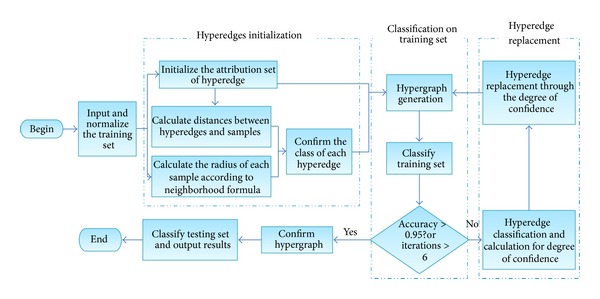
The flow chart of algorithm.

The flow chart of the algorithm is shown in Figure 4. Then, one analyzes each part of the flow chart of the remaining section specifically as follows.

### 3.1. Hyperedge Initialization

Hyperedges are generated based on the samples, which reserve the real distribution of the sample set and thereby provide a foundation for hyperedge selection. Meanwhile, one can change some attribute values while generating hyperedge. Thus, more decision rules are generated for sample classification, which can improve the accuracy of sample classification to some extent.

In this paper, attribution set of hyperedges is *C* (namely, the universal set of attributions for samples); that is to say, a hyperedge is exactly a sample and denoted by a small dot in the figure. In hyperedge initialization, we can assign a value on each attribute and determine the classification of each hyperedge.

In order to process imbalanced dataset, two classes will be considered in the following definitions.

**Definition 10 .**

Given *G* = 〈*X*, *E*〉, for arbitrary *x* ∈ *X*, assume *D*(*x*)∈{*P*, *N*}, where *P* denotes minority decision and *N* denotes majority decision. Then sets of decision *P* and *N* decision in hyperedge set are, respectively, defined as

(10) $$\begin{array}{c} nX\left(P\right)=\left\{x\;|\;D\left(x\right)=P,x\in E\right\},\\ nX\left(N\right)=\left\{x\;|\;D\left(x\right)=N,x\in E\right\}. \end{array}$$

So the degree of imbalance for *E* is defined as

(11) $$\begin{array}{c} inbanX=\frac{\left|nX\left(N\right)\right|}{\left|nX\left(P\right)\right|}. \end{array}$$

In this paper, the process of hyperedge generation consisted of two stages: attribution inheritance and class confirmation.

**Figure 5:**
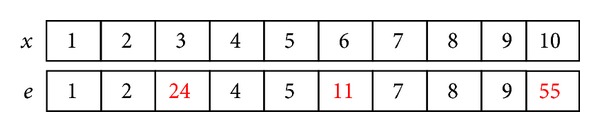
Attribution inheritance.

(1) Attribution inheritance: hyperedge and sample have the same attribution set, and the attribute values of the hyperedge are assigned partly based on the sample. One selects 7/10 of all attributes of one hyperedge which are selected randomly and they inherit the corresponding attribution value of the sample. The remaining attribute values are generated randomly in the range of the corresponding attribute values of the sample. In Figure 5, *x* is a sample and *e* is a hyperedge.

**Definition 11 .**

Given *G* = 〈*X*, *E*〉, for arbitrary *e* ∈ *E*, the majority sample set and the minority sample set related to hyperedge *e* in the sample set are defined as

(12) $$\begin{array}{c} inN\left(e\right)=\left\{x\;|\;e\in \delta \left(x\right),D\left(x\right)=N,x\in X\right\},\\ inP\left(e\right)=\left\{x\;|\;e\in \delta \left(x\right),D\left(x\right)=P,x\in X\right\}. \end{array}$$

(2) Class confirmation: the class of a hyperedge is confirmed by the whole dataset. There are two cases as follows.

- According to formula (11), if (|*in* *N*_*B*_(*e*)|/|*in* *P*_*B*_(*e*)|) ≥ *in* *b* *a* *n* *X*, then *D*(*e*) = *N*;
- otherwise, generate a random number *r* in [0, 1]. If *r* ≥ (|*X*_*N*_|/(|*X*_*P*_| + |*X*_*N*_|)) (*X*_*N*_, *X*_*P*_, respectively, represent majority sample set and minority sample set), then *D*(*e*) = *N*; else, *D*(*e*) = *P*.

### 3.2. Classification on Training Set

One uses the generated hyperedge set to classify the training set. Through analyzing the classification result, one can know the classification accuracy of hyperedge set and determine whether to replace the hyperedge for the hyperedge set or not. By repeating the process of training sample classification and hyperedge replacement, we can make the distribution of hyperedge set approach the real distribution of training sample set gradually.

**Definition 12 .**

Given *G* = 〈*X*, *E*〉, the majority hyperedge set and minority hyperedge set in neighborhood of sample *x* are defined as

(13) $$\begin{array}{c} ne\left(x,N\right)=\left\{e\;|\;D\left(e\right)=N,e\in \delta \left(x\right)\right\},\\ ne\left(x,P\right)=\left\{e\;|\;D\left(e\right)=P,e\in \delta \left(x\right)\right\}. \end{array}$$

One should consider the factor when we use neighborhood hypergraph to classify the samples: (1) the amounts of majority hyperedge and minority hyperedge in the neighborhood of a sample; (2) the degree of the imbalance of hyperedge set. Combining with the above, one presents the classification method.

Given sample set *X*, for arbitrary *x* ∈ *X* and attribution set *B*⊆*C*,

(1) if *ne*(*x*, *N*)/*ne*(*x*, *P*) ≥ *in* *b* *a* *n* *E* ^2^, then *D*(*x*) = *N*;

(2) if *ne*(*x*, *N*)/*ne*(*x*, *P*) < *in* *b* *a* *n* *E* ^2^, then *D*(*x*) = *P*.

One uses the classification rules above to classify the training set. If the accuracy is higher than 0.95, one can output the hyperedge set. Otherwise, the hyperedge replacement operation should be adopted (see Section 3.3).

In experimental evaluation, we conclude that *in* *b* *a* *n* *E* ^2^ is a good choice to enhance the accuracy of minority class, while *in* *b* *a* *n* *E* is a poor one to classify the minority class samples.

### 3.3. Hyperedge Replacement

In the process of hyperedge initialization, one generates part of the attribute values randomly. As a result, some of hyperedges are not suitable for sample classification. In order to acquire better performance, one should replace the poor hyperedges by generating new hyperedges, namely, hyperedge replacement.

The algorithm divided hyperedge set into upper approximation, lower approximation, boundary region, and negative region. The confidence degree of hyperedges in lower approximation is 1. The confidence degree of hyperedges in boundary region is between 0 and 1. Hyperedges whose confidence degree is 0 belong to negative region. Hyperedges in lower approximation are all retained because they are very helpful for classification. On the contrary, since hyperedges in negative region are counteractive for classification, they will be replaced. For the hyperedges in boundary region, they will be dealt with by a threshold *λ*. When the confidence degree of a hyperedge is less than *λ*, it will be replaced. Through the above, one can enhance the pertinence and validity of hyperedge replacement.

It is composed of three steps.

1. Set the confidence degree threshold of each hyperedge (in this paper *λ* ∈ (0.75,1)).
2. Find out those hyperedges whose confidence degree is under the threshold from the hyperedge set.

According to Definitions 7 and 8, one can calculate the confidence degree of each hyperedge following the three cases below (*e*_*i*_ denotes a hyperedge).

**Case 1 .**

**Figure 6:**
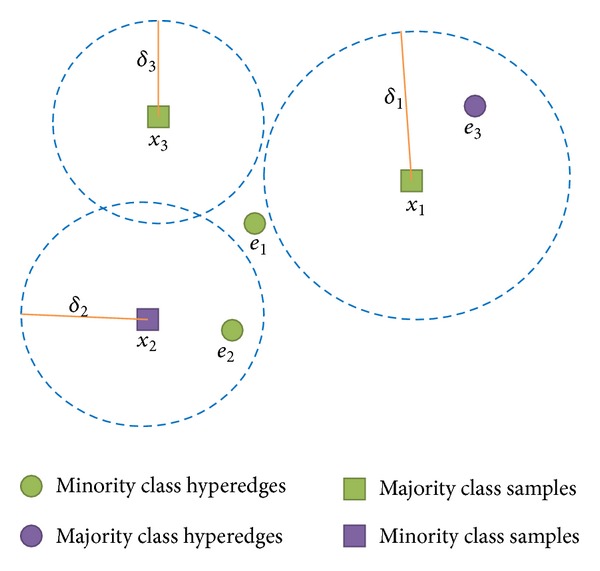
An example of situation 1.

If *in*_*B*_(*e*) = *ϕ*, then *e*_1_ is not in any neighborhood of samples, as shown in Figure 6.

In this case, one can assume that *e*_1_ is in the overlapped neighborhood of the nearest five samples. Then the confidence degree of *e*_1_ can be calculated according to Formula (6).

**Case 2 (Conf_*B*_(*e*) ≥ *λ*).**

It means that samples surrounding *e*_1_ have the same class with *e*_1_. Thus *e*_1_ is helpful for the sample classification and should remain.

**Case 3 (0 ≤ Conf_*B*_(*e*) < *λ*).**

**Figure 7:**
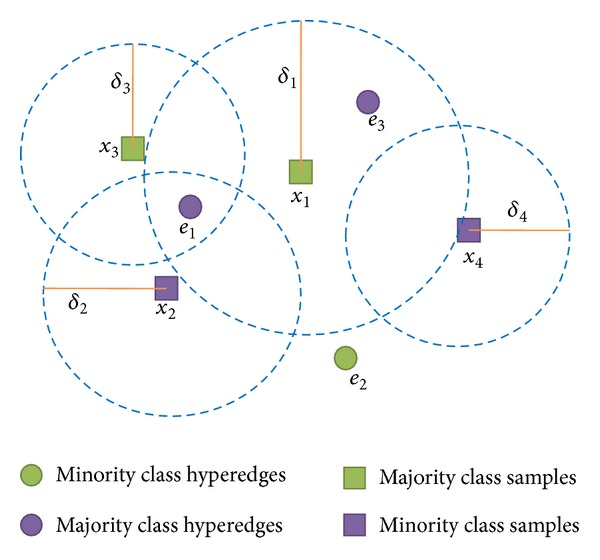
Hyperedges whose confidence degree is under *λ*.

Now, let us give an example below to explain the situation (see Figure 7).

According to Formula (4) and Definition 6, we know *in*_*C*_(*e*_1_) = {*x*_1_, *x*_2_, *x*_3_}, *in*_*C*_(*e*_2_) = *ϕ*, and *in*_*C*_(*e*_3_) = {*x*_1_}. Then according to formula (8), Conf_*B*_(*e*_1_) = 1/3 and Conf_*B*_(*e*_3_) = 0 can also be calculated. However, as it is difficult to determine the nearest five samples surrounding *e*_2_, we cannot calculate the confidence degree of *e*_2_.

This kind of hyperedges has the same class with few samples surrounding them, which results in the poor effect on classification. Thus, they should be replaced.

(3) Generate new hyperedge and hyperedge replacement.

One selects a hyperedge *e*_*i*_ from the hyperedge set which should be replaced and generates a sample *x*. Then a new hyperedge *e*_*j*_ is initialized by using *x*. After that, one can replace *e*_*i*_ with *e*_*j*_. Repeat the process above until no hyperedge needs to be replaced.

### 3.4. Neighborhood Hypergraph Algorithm

In this paper, sample classification and hyperedge replacement are based on the neighborhood radius of sample. According to Definition 1, the computational formula of the neighborhood radius of sample, denoted by *δ*, is as follows [34]:

(14) $$\begin{array}{c} \delta =\min\left(\Delta \left(x_{i},s\right)\right)+w\times \text{range}\left(\Delta \left(x_{i},s\right)\right),\;0\leq w<1, \end{array}$$

where *x*_*i*_  (*i* = 1,2,…, *n*) is a training sample, min⁡(Δ(*x*_*i*_, *s*)) denotes the minimal value of distance between *x*_*i*_ and the remaining samples excluding *s*, and range(Δ(*x*_*i*_, *s*)) denotes the value domain of Δ(*x*_*i*_, *s*).

Here we give the N-HyperGraph (see Algorithm 1).

There are two main parameters in the algorithm: (1) the radius of neighborhood *w*; (2) threshold of the confidence degree *λ*. The former is important to control the number of hyperedges. The number is increasing with the increasing of *w*. The latter is vital to ensure the quality of hyperedges. The higher *λ* is, the better hyperedges can be obtained. Of course, when the *λ* is too big, there is no sense that almost all the hyperedges will be replaced.

## 4. Experimental Designing and Analysis

### 4.1. Datasets

In order to test the proposed algorithm in this paper, one selects 18 UCI datasets which are downloaded from the machine learning data repository, University of California, at Irvine. The imbalanced rate is from 1.37 to 28.10. There are seven multiclass datasets and eleven two-class datasets. Multiclass datasets are modified to obtain two-class imbalance problems, by the union of one or more classes of the minority class and the union of one or more of the remaining classes which are labeled as the majority class. For the missing values, if they are continuous features, we fill them with average values; if they are nominal features, we fill them with values that appear most frequently. The datasets are outlined in Table 1 and sorted by imbalanced rates from low to high.

### 4.2. Experimental Evaluation in Imbalanced Domains

The traditional evaluation usually uses Confusion Matrix, showed in Table 2, where TP means the number of positive samples that are classified into positive, TN means the number of negative samples that are classified into negative, FN means the number of positive samples that are misclassified, and FP means the number of negative samples that are misclassified.

From Table 2, one could get some useful evaluation as follows.

Precision = TP/(TP + FP); Recall = TP/(TP + FN); *F*-Value = 2RP/(R + P), where R and P refer to Recall and Precision, respectively.

There are three evaluations as the formulas called Precision, Recall, and *F*-value. Precision (also called positive predictive value) is the fraction of retrieved instances that are relevant, while Recall (also known as sensitivity) is the fraction of relevant instances that are retrieved. From the previous formulas, we can decrease FP to increase Precision and increase TP to increase Recall. But in fact they conflicted. So we use the *F*-value to consider them comprehensively. Only when Precision and Recall are both higher, *F*-value will be higher.

Another appropriate metric that could be used to measure the performance of classification over imbalanced datasets is the receiver operating characteristic (ROC) graphics [37]. In these graphics, the tradeoff between the benefits (TP) and costs (FP) can be visualized, and it acknowledges the fact that the capacity of any classifier cannot increase the number of true positives without also increasing the false positives. The area under the ROC curve (AUC) [38] corresponds to the probability of correctly identifying which of the two stimuli is noise and which is signal plus noise. AUC provides a single number summary of the performance of learning algorithms.

### 4.3. Experimental Methods

In order to evaluate the performance of N-HyperGraph in this paper, one compares it with some other algorithms in related literatures: SVM and J48 (C4.5) [39] implemented on Weka [40], NRSBoundary-SMOTE [12]+C4.5, SMOTE-RSB∗ [29]+C4.5, CS-EN-HN [7], and N-HyperGraph implemented by Java programming language. Among these algorithms, SVM classifies the source datasets directly. J48 (C4.5) classifies those datasets after oversampling by NRSBoundary-SMOTE and SMOTE-RSB. The oversampling rate is 100%. CS-EN-HN is an ensemble method of cost-sensitive hypernetwork. It deals with datasets which are discretized by optimal class-dependent discretization (OCDD) [41]. Ones take the value of win the range [0.001–0.6] in N-HyperGraph. We use 10-fold cross validation as validation method.

### 4.4. Experimental Results and Analysis

Contrastive experiment results on Precision, Recall, *F*-value, *G*-means, and AUC among each algorithm are shown in Table 3 to Table 7.

**Figure 8:**
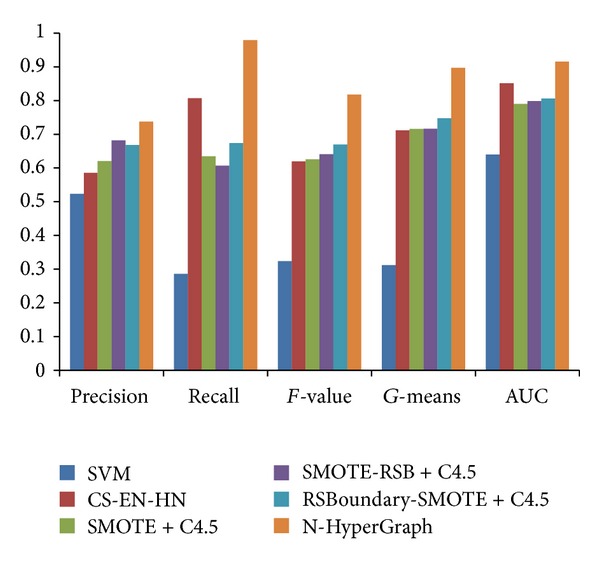
The average value of each indicator.

In order to view the performance on 5 algorithms, the average accuracies of different indicator of 5 algorithms are showed in Figure 8.

Tables 3, 4, 5, 6, and 7 point out that N-HyperGraph has high performance than other four algorithms on most datasets mentioned above. The average result of Precision is enhanced to 0.7374 while it ranges between 0.5233 and 0.6816 for the other four algorithms. The average value of Recall increases to 0.9785 while it changes between 0.2858 and 0.8068. The average value of *F*-value is up to 0.8173 while it ranges between 0.3235 and 0.6695. Meanwhile, the average values of *G*-means and AUC increase to 0.8968 and 0.9152 while their values take from 0.3118 to 0.7475 and 0.6398 to 0.8509, respectively.

We can find out from Tables 3–7 and Figure 8 that the Precision performance is unsteady for the proposed algorithms N-HyperGraph. As it is mentioned before, the process of hyperedge initialization is based on the degree of imbalance of training set. The generation of hyperedges depends on the imbalanced degree, which results in the fact that the generated hyperedges incline to the minority sample. Thus, the proposed algorithm is not steady on Precision. But, it can work better than SVM and J48 (C4.5) for Recall, *F*-value, *G*-means, and AUC.

In total, the experimental results of N-HyperGraph are better than all of the other algorithms. Since the rough set theory is used in N-HyperGraph, it is more efficient to process the uncertain samples, especially in boundary region of hyperedge set. What is more, weights are calculated through the neighborhood rough set model; thus it makes more hyperedges involve in the class decision of a hyperedge, improving the accuracy. Due to the two aspects above in the proposed algorithm, the results of classification are improved.

As SMOTE oversamples all minority class samples, it decreases the decision space of majority class. Although it can improve Recall of minority class, many majority class samples will be misclassified as minority class, thereby resulting in the decreasing of Precision. SMOTE-RSB filters the synthetic samples more strictly than SMOTE, because few synthetic samples are generated when datasets are highly imbalanced. Thus, compared with SMOTE, its improvement is not obvious. RSBoundary-SMOTE takes neighborhood rough set into consideration and emphasizes resampling for minority class samples which belong to boundary region and thus improves the *F*-value. However, N-HyperGraph replaces hyperedges repetitively according to neighborhood rough set. The distribution of hyperedge set draws near to the true distribution of samples gradually, which makes a more obvious improvement on classification performance. Besides, since CS-EN-HN can just deal with discrete data, too much information loss of data makes its performance worse than N-HyperGraph.

## 5. Conclusion

In this paper, one proposed a new algorithm based on hypernetwork called N-HyperGraph to solve the problem of classifying imbalance dataset. At first, hyperedge set is divided according to rough set theory. Then, some poor hyperedges are replaced by combining with the imbalanced degree, in order to improve the accuracy. The experimental results on 18 UCI datasets with different degree of imbalance show that the classification result of the proposed algorithm N-HyperGraph improves obviously in contrast with another four algorithms. However, the algorithm N-HyperGraph will cost much time, due to calculating the distance between hyperedge and sample. Thus, how to reduce the running time of the algorithm is our future work.

## Figures and Tables

**Algorithm 1:**
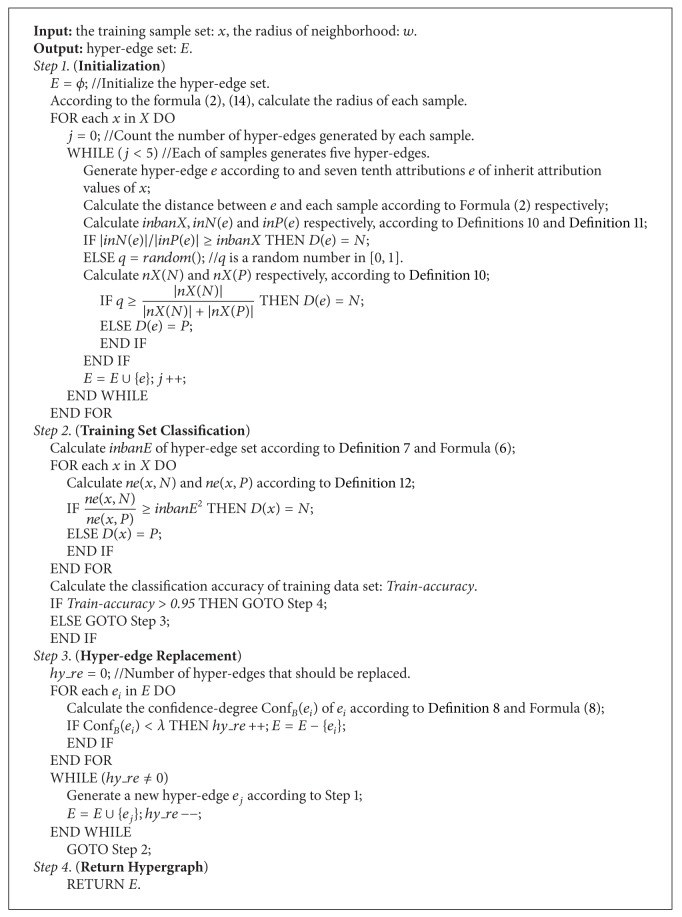
Neighbor hypergraph (N-HyperGraph).

**Table 1:** Data description.

Dataset	Size	Attribute	Class label (minority : majority)	Class distribution
Bupa	345	6C	01 : 02	145/200
Colic	368	7C 15N	No : yes	136/232
Reprocessed	294	13C	01 : 00	106/188
Machine	209	7C	Others : 2	74/135
Labor	57	8C 8N	Bad : good	20/37
Tic	958	9N	Negative : positive	332/626
Iris	150	4C	Iris-virginica : others	50/100
Seed	210	7C	02 : others	70/140
Vc	310	6C	Normal : Abnormal	100/210
Glass	214	9C	01, 02 : others	68/146
Haberman	306	3C	02 : 01	81/225
Transfusion	748	4C	01 : 00	178/570
Abalone (7 : 15)	494	7C 1N	15 : 07	103/391
Balance-scale	625	4C	B : others	49/576
Abalone (9 : 18)	731	7C 1N	18 : 9	42/689
Yeast (POX : CTY)	483	8C	POX : CYT	20/463
Car	1728	6N	Good : others	69/1659
Yeast (ME2 : others)	1484	8C	ME2 : others	51/1433

C: continuous, N: nominal.

**Table 2:** Confusion matrix.

	Predict to positive	Predict to negative
Positive	TP	FN
Negative	FP	TN

**Table 3:** Precision.

Dataset	SVM	CS-EN-HN	SMOTE + C4.5	SMOTE-RSB∗ + C4.5	NRSBoundary-SMOTE + C4.5	N-HyperGraph
Bupa	**0.8571**	0.6827	0.5663	0.6581	0.5614	0.5497
Colic	0.6857	0.5887	0.7391	0.8103	0.7687	**0.9151**
Reprocessed	0.0000	0.6344	0.7030	**0.7187**	0.6979	0.5558
Machine	0.0000	0.7542	0.8250	0.8873	**0.9041**	0.9025
Labor	0.9411	**1.0000**	0.6667	0.6667	0.8421	0.7733
Tic	0.9908	0.6534	0.6882	0.8044	0.8100	**0.9942**
Iris	**0.9245**	0.5061	0.9057	0.8703	0.8888	0.8310
Seed	0.9295	**0.9750**	0.9577	0.9577	0.9577	0.8614
Vc	0.0000	0.6133	0.6696	**0.7529**	0.6695	0.4842
Glass	0.8775	**1.0000**	0.6933	0.8214	0.7428	0.8652
Haberman	0.4999	0.2334	0.4516	0.4590	0.4948	**0.7433**
Transfusion	0.4186	0.3139	0.4722	0.5299	0.5000	**0.7116**
Abalone (7 : 15)	0.7951	0.7552	0.8056	0.8155	0.8100	**0.9413**
Balance-scale	0.0000	0.1605	0.0000	0.0000	0.0000	**0.4942**
Abalone (9 : 18)	0.0000	0.3910	0.4167	0.4347	0.5000	**0.9500**
Yeast (POX : CTY)	**1.0000**	0.6371	0.6000	0.9268	0.7736	0.7000
Car	0.5000	0.5100	0.6849	0.6849	**0.6857**	0.6731
Yeast (ME2 : others)	0.0000	0.1272	0.3214	**0.4706**	0.4200	0.3265

Average	0.5233	0.5853	0.6204	0.6816	0.6682	**0.7374**

*w* = 0.001 to 0.6.

**Table 4:** Recall.

Dataset	SVM	CS-EN-HN	SMOTE + C4.5	SMOTE-RSB∗ + C4.5	NRSBoundary-SMOTE + C4.5	N-HyperGraph
Bupa	0.0413	0.7856	0.6483	0.5310	0.6621	**0.8405**
Colic	0.1764	0.6531	0.7500	0.6912	0.7574	**1.0000**
Reprocessed	0.0000	0.5999	0.6698	0.6509	0.6320	**1.0000**
Machine	0.0000	0.8404	8919	0.8514	0.8919	**0.9732**
Labor	0.8000	0.5000	0.5000	0.7000	0.8000	**0.9000**
Tic	0.6536	**1.0000**	0.7711	0.7680	0.7319	**1.0000**
Iris	0.9800	0.5000	0.9600	0.9400	0.9600	**1.0000**
Seed	0.9428	0.9428	0.9714	0.9714	0.9714	**1.0000**
Vc	0.0000	0.9500	0.7700	0.6400	0.7900	**1.0000**
Glass	0.6323	**0.9790**	0.7647	0.6764	0.7647	0.9000
Haberman	0.0246	0.7732	0.5185	0.3457	0.5926	**1.0000**
Transfusion	0.1011	0.9117	0.4775	0.3989	0.5000	**1.0000**
Abalone (7 : 15)	0.6407	**1.0000**	0.8447	0.8155	0.7864	**1.0000**
Balance-scale	0.0000	0.5500	0.0000	0.0000	0.0000	**1.0000**
Abalone (9 : 18)	0.0000	0.8666	0.3571	0.4347	0.3809	**1.0000**
Yeast (POX : CTY)	0.1372	0.8000	0.4500	0.4751	0.8039	**1.0000**
Car	0.0145	**1.0000**	0.7246	0.7246	0.6956	**1.0000**
Yeast (ME2 : others)	0.0000	0.8700	0.3529	0.3137	0.4118	**1.0000**

Average	0.2858	0.8068	0.6346	0.6071	0.6740	**0.9785**

*w* = 0.001 to 0.6.

**Table 5:** *F*-value.

Dataset	SVM	CS-EN-HN	SMOTE + C4.5	SMOTE-RSB∗ + C4.5	NRSBoundary-SMOTE + C4.5	N-HyperGraph
Bupa	0.0789	**0.7193**	0.6045	0.5878	0.6076	0.6422
Colic	0.2807	0.5688	0.7445	0.7460	0.7630	**0.9530**
Reprocessed	0.0000	0.5374	0.6698	0.6831	0.6633	**0.7118**
Machine	0.0000	0.8404	0.8571	0.8690	0.8980	**0.9242**
Labor	**0.8648**	0.6666	0.5714	0.6829	0.8205	0.8171
Tic	0.7876	0.7900	0.7273	0.7858	0.7689	**0.9926**
Iris	**0.9514**	0.4835	0.9320	0.9038	0.9230	0.9045
Seed	0.9361	0.9545	**0.9645**	**0.9645**	**0.9645**	0.9209
Vc	0.0000	**0.7295**	0.7163	0.6919	0.7248	0.6501
Glass	0.7350	**0.9890**	0.7273	0.7419	0.7536	0.8808
Haberman	0.0470	0.3475	0.4828	0.3944	0.5393	**0.8396**
Transfusion	0.1628	0.4582	0.4749	0.4551	0.5000	**0.8119**
Abalone (7 : 15)	0.7096	0.8595	0.8246	0.8155	0.7980	**0.9683**
Balance-scale	0.0000	0.2326	0.0000	0.0000	0.0000	**0.6447**
Abalone (9 : 18)	0.0000	0.5071	0.3846	0.3076	0.4324	**0.9709**
Yeast (POX : CTY)	0.2413	0.6493	0.5143	**0.8261**	0.7885	0.8066
Car	0.0282	0.6728	0.7042	0.7042	0.6906	**0.7976**
Yeast (ME2 : others)	0.0000	0.1924	0.3364	0.3765	0.4158	**0.4750**

Average	0.3235	0.6191	0.6252	0.6409	0.6695	**0.8173**

*w* = 0.001 to 0.6.

**Table 6:** *G*-means.

Dataset	SVM	CS-EN-HN	SMOTE + C4.5	SMOTE-RSB∗ + C4.5	NRSBoundary-SMOTE + C4.5	N-HyperGraph
Bupa	0.2026	**0.7456**	0.6441	0.6518	0.6433	0.5592
Colic	0.4008	0.5828	0.7740	0.7910	0.8100	**0.9687**
Reprocessed	0.0000	0.5973	**0.7503**	0.7466	0.7311	0.7247
Machine	0.0000	0.8371	0.8941	0.8949	0.9196	**0.9377**
Labor	0.8000	0.7071	0.6576	0.7534	**0.8574**	0.8232
Tic	0.8015	0.8466	0.7926	0.8318	0.8156	**0.9959**
Iris	0.4427	0.5112	**0.9550**	0.9349	0.9499	0.9427
Seed	0.6473	0.9623	**0.9750**	**0.9750**	**0.9750**	0.9511
Vc	0.0000	0.7729	0.7941	0.7589	**0.8021**	0.6831
Glass	0.7141	**0.9892**	0.8026	0.7938	0.8187	0.8897
Haberman	0.2957	0.4884	0.6332	0.5431	0.6808	**0.9005**
Transfusion	0.1628	0.4582	0.6308	0.5956	0.6496	**0.8119**
Abalone (7 : 15)	0.6626	0.9565	0.8940	0.8808	0.8649	**0.9909**
Balance-scale	0.0000	0.6016	0.0000	0.0000	0.0000	**0.9428**
Abalone (9 : 18)	0.0000	0.8521	0.5884	0.4833	0.6100	**0.9978**
Yeast (POX : CTY)	0.3704	0.7088	0.6665	0.8552	0.8630	**0.9879**
Car	0.1195	0.9790	0.8453	0.8453	0.8285	**0.9888**
Yeast (ME2 : others)	0.0000	0.5086	0.5861	0.5566	0.6352	**0.9485**

Average	0.3118	0.7113	0.7158	0.7162	0.7475	**0.8968**

*w* = 0.001 to 0.6.

**Table 7:** AUC.

Dataset	SVM	CS-EN-HN	SMOTE + C4.5	SMOTE-RSB∗ + C4.5	NRSBoundary-SMOTE + C4.5	N-HyperGraph
Bupa	0.5181	**0.8928**	0.6468	0.6652	0.6401	0.6427
Colic	0.5645	0.8265	0.7960	0.7855	0.8102	**0.9699**
Reprocessed	0.5000	0.6750	**0.7896**	0.7565	0.7817	0.7662
Machine	0.5000	0.9201	0.9199	0.9359	0.9430	**0.9437**
Labor	0.8864	0.7500	0.7500	0.7655	0.8243	**0.8875**
Tic	0.8252	**1.0000**	0.8638	0.8941	0.8848	0.9959
Iris	**0.9700**	0.7500	0.9408	0.9197	0.9468	0.9450
Seed	0.9535	0.9780	0.9730	**0.9782**	**0.9782**	0.9535
Vc	0.5000	**0.9750**	0.8107	0.8380	0.8277	0.7381
Glass	0.7956	**0.9894**	0.8442	0.8640	0.8637	0.9400
Haberman	0.5079	0.5866	0.6255	0.6174	0.6636	**0.9186**
Transfusion	0.5286	0.8558	0.6813	0.7007	0.7048	**0.9114**
Abalone (7 : 15)	0.7986	**1.0000**	0.8901	0.9046	0.8627	0.9910
Balance-scale	0.5000	0.6256	0.5000	0.5000	0.5000	**0.9452**
Abalone (9 : 18)	0.6611	0.9333	0.7294	0.6514	0.7065	**0.9978**
Yeast (POX : CTY)	0.5000	0.8999	0.6881	0.8685	0.8762	**0.9881**
Car	0.5069	0.9792	0.9775	0.9775	**0.9911**	0.9888
Yeast (ME2 : others)	0.5000	0.6790	0.7894	0.7412	0.6916	**0.9504**
Average	0.6398	0.8509	0.7898	0.7980	0.8054	**0.9152**

*w* = 0.001 to 0.6.
